# Sport, Sexual Violence and the Law: A Feminist Critique and Call to Action

**DOI:** 10.1007/s40318-022-00230-5

**Published:** 2022-11-11

**Authors:** Jason Haynes

**Affiliations:** 1 University of Birmingham, Birmingham Law School, Birmingham, UK; 2 Birmingham, UK

**Keywords:** Sexual Violence, Feminism, Law

## Abstract

Sport is a microcosm of society. In this connection, in as much as there are reasons to celebrate individual athletic prowess and the undeniable contribution of sport to the maturation of communities, sport, like the broader society, contends with many ills, not least sexual violence. Although various sporting organizations and governments have, in the last 2 decades, adopted legislative instruments and Codes of Ethics and established various institutional mechanisms to combat the scourge of sexual violence, sport remains a hot bed for sexual violence, intimidation, reprisals and indignity in many jurisdictions. This article accordingly interrogates, from a Feminist Perspective, recently decided cases to illustrate how sexual violence committed against women and girls in the sporting context reflects a broader deeply entrenched system of patriarchy, characterized by a culture of silence, indifference, and abuse of authority. It concludes by calling on all concerned in the governance, administration, and practice of sport to redouble their efforts to address the growing problem of sexual violence in sport.

## Introduction

In 2017, three United States former gymnasts, Jeanette Antolin, Jessica Howard, and Jamie Dantzcher, gave an interview in which they accused Larry Nassar, the long-standing national medical coordinator for USA Gymnastics, of sexual violence.^1^ The women alleged that they, along with hundreds of other women and girls, suffered at the hands of Nassar, whom they indicated created an ‘emotionally abusive environment.’^2^ Another woman, Olympic Gold Medalist McKayla Maroney, using the #MeToo hashtag on Twitter, also brought attention to Nassar having repeatedly molested her from the age of 13 years until she retired from the sport 8 years later.^3^ She later filed a claim against USA Gymnastics for covering up the sexual violence that she experienced, but this was subsequently withdrawn after she entered into a $1.25 million settlement agreement.^4^ Other women complained about Nassar having used his position to groom vulnerable girls who were under his supervision.^5^ Nassar pleaded guilty for, among other things, first-degree criminal sexual conduct with minors under the age of 16, effectively admitting to having molested 7 girls. In 2018, he received a practical life sentence for the offense of sexual assault against minors.^6^

In the wake of his conviction, Nassar’s victims began filing civil lawsuits in an attempt to hold USA Gymnastics and Michigan State University, to which Nassar was formerly employed, accountable for allegedly enabling the abuse perpetrated against them and for negligently allowing the abuse to occur over several years.^7^ They also sued the United States Olympic & Paralympic Committee on similar grounds. The prospect of being held liable on account of the institution of several tortious claims ultimately resulted in USA Gymnastics filing for bankruptcy in a US court.^8^

The Nassar saga catalyzed a series of responses from the US Congress, which appointed a Subcommittee to investigate the potential failings of the parties involved.^9^ The Committee, having held four hearings on sexual violence in amateur sports, and having interviewed dozens of athletes and survivors, coaches, parents, advocates, United States Olympic Committee (USOC), USA Gymnastics (USAG) and National Governing Body (NGB) officials, SafeSport officials, law enforcement, and others, found that:

- Nassar was able to abuse over 300 athletes over two decades because of ineffective oversight by USAG and USOC;
- USAG and USOC failed to uphold their statutory purposes and duties to protect amateur athletes from sexual, emotional, or physical abuse;
- USOC, USAG, MSU, and the FBI had opportunities to stop Nassar but failed to do so; and
- USAG and USOC knowingly concealed abuse by Nassar, leading to the abuse of dozens of additional amateur athletes during the period beginning the summer of 2015 and ending in September 2016.

Against this backdrop, the Committee made several recommendations aimed at addressing the concerns raised by the Nassar debacle, namely:

- Improving amateur athletes’ representation within the U.S. Olympic and Paralympic Committee and National Governing Bodies;
- Enhancing the independence of the Office of the Athlete Ombudsman whose aim it is to advise athletes;
- Imposing procedural limitations on interactions between amateur athletes that are minors and adults (besides their guardians);
- Expanding statutory retaliation protection against parties who report sexual violence;
- Publishing on the U.S. Center for SafeSport website a comprehensive list of individuals who are barred from the USOPC or an NGB;
- Developing training materials on sexual violence for specific audiences, including coaches, trainers, doctors, young children, adolescents, adults and mentally disabled individuals;
- Ensuring the independence of the U.S. Center for SafeSport from influence of USOPC, NGBs, or other entities;
- Codifying the requirement for the U.S. Center for SafeSport to immediately report allegations of child abuse to law enforcement;
- Statutorily requiring SafeSport and its staff to not take any action that will notify an alleged perpetrator of abuse of an amateur athlete of any ongoing investigation or accusation unless law enforcement authorized them to do so, or unless the Center has reason to believe an imminent hazard will result from failing to so notify the alleged perpetrator; and
- Requiring SafeSport to establish procedures to prohibit retaliation by NGBs or Paralympic sports organizations where an amateur athlete has made a report related to abuse, including emotional and physical abuse, or sexual violence.

Interestingly, although the *Protecting Young Victims from Sexual violence and Safe Sport Authorization Act* established the United States Center for SafeSport in 2017 as an independent entity to investigate reports of abuse and to protect athletes from abuse in the United States Olympic movement, its legitimacy and effectiveness was almost immediately called into question against the background of the Nasser saga.

A year after the Subcommittee’s report was published, the *Empowering Olympic and Amateur Athletes Act* of 2019 was passed, which came into effect in 2020. Among other things, the Act establishes mechanisms to ensure that the Olympic movement protects survivors of sexual violence by requiring the U.S. Olympic and Paralympic Committee to establish clear procedures and reporting requirements that will safeguard athletes who come forward; and by imposing clear responsibilities to protect athletes who participate in sports programs administered by National Governing Bodies.

While the USA’s *Empowering Olympic, Paralympic, and Amateur Athletes Act* represents a powerful response to the problem of sexual violence in sport, it is not the only piece of legislation in a broader global context that aims to protect the interests of minor athletes. Across the Atlantic, the United Kingdom enacted the *Safeguarding Vulnerable Groups Act* in 2006. This Act, while not a *sui generis* piece of sporting legislation, envisages that a person will be placed on ‘the children's barred list’ and thereby prevented from working with children if he/she *may* harm a child; cause a child to be harmed; put a child at risk of harm; attempt to harm a child; or incite another to harm a child.

The UK’s enactment of the *Safeguarding Vulnerable Groups Act,* while a welcome step in the right direction, has not, however, resulted in the elimination of sexual violence against athletes in that jurisdiction.^10^ In fact, in June 2022, Anne Whyte QC, conducted an independent investigation, commissioned by Sport England and UK Sport, following allegations of mistreatment within the sport of gymnastics. The resulting report, ‘The Whyte Review’,^11^ found that in over 30 submissions from club owners, coaches, gymnasts, parents and others within gymnastics, there was evidence of sexual violence. The issues raised ranged from grooming and sexual assault to sexual remarks and inappropriate relationships between coaches and gymnasts. Other examples of problematic behaviours reported to Whyte were gymnasts being tickled, touched on the bottom unnecessarily during gymnastic moves, threatened with being kissed as a punishment for not following instructions and sexualized comments of a personal nature.

In the broader European context, the European Commission reports that the prevalence of sexual violence in sport in Europe is between 5% and 17%, with most of those subject to such conduct being women and girls.^12^ More specifically, in France, it was reported in 2021 that, after a year-long, nationwide French effort to uncover and combat sexual violence in sports, more than 400 coaches, teachers and others suspected of abuse or covering it up were identified.^13^ The report noted that most of the victims were under 15, and that sixty people were at the time facing criminal proceedings, while more than 100 were temporarily or permanently removed from their posts. The accusations were reported in respect of nearly 48 sporting federations. Of those accused, 96% were men, while of the victims, 83% were women or girls, and 63% were under age 15. The fact-finding probe was launched in February 2020 after 10-time French skating champion Sarah Abitbol said in a book that she was raped by coach Gilles Beyer from 1990 to 92, when she was a teen.^14^

Meanwhile, in Germany, one recent study found that, of the 1529 German elite athletes over 16 years of age from 128 different sports surveyed, 37.6% reported that they had experienced at least one incident of sexual violence in organized sport, with 11.2% reporting a severe form of sexual violence.^15^ The report concluded that female athletes were affected significantly more often than male athletes by sexual violence.

Further afield, at the global level, there is concrete evidence that sexual violence occurs in various sporting disciplines. One recent large-scale study, published in the British Journal of Sports Medicine, for example, estimated sexual violence in sport globally to be between 2% and 48%. The authors, Saul Marks, Margo Mountjoy and Madalyn Marcus, noted that sexual violence is higher in elite sport, as the ‘higher the athlete is on the sporting talent ladder, the greater the risks of [them] being sexually exploited.’^16^ The authors further explained that persons engaging in sports where there is early specialization are at greater risk of being subject to sexual violence, especially in sports where intensive talent identification happens around puberty. They consider that this might be on account of the fact of the increased dependence on coaches to reach performance goals.

Beyond statistical references, the authors provide startling insights into the relationship dynamics between coaches and athletes in cases in which sexual violence is alleged. They noted that coaches, as perpetrators, are often in stronger positions of power than other members of the athletes’ entourage, although they recognized that peer athletes tend to harass their peers more than coaches do. The authors usefully identified the primary sites of exploitation as being in locker rooms, coaches’ cars, and homes, and on trips away to isolated locations.

The authors’ work also provides startling insights into the negative consequences associated with sexual violence in sport. More pointedly, they report that many survivors complain of experiencing headaches, lethargy, sleep disturbances, weight fluctuations and poorer general health satisfaction, and that these athletes tend to have poorer health outcomes in respect of their general health, gastrointestinal health, gynecological or reproductive health, pain, cardiopulmonary symptoms, and obesity. The psychological consequences are equally startling; many survivors complain of bed wetting, fatigue, acting out behaviours, risky sexual activities that often lead to the transmission of sexually transmitted infections, aggressive behaviour, as well as self-harm or self-abuse, including excessive dieting or bingeing.

The authors also provide important insights into how the power dynamics between coaches and athletes manifest into sexual violence. They note that coaches tend to target potentially vulnerable athletes and begin a friendship with them. Through this friendship, the coach builds a relationship of trust by making the athlete feel special by the giving of rewards or gifts. The coaches then develop further control and loyalty often through refusing the athlete access to significant others, friends, and support services. Implicated coaches thereafter continue to build and secure secrecy by ensuring that sexual boundaries are blurred.

Similar sentiments have been articulated by Sonja Gaedicke et al., who contend that ‘intense relationships between coaches and athletes seem to be a prerequisite for promoting young athletes' success in sport. At the same time, such close relationships carry risks for negative dependencies, misuse of trust, and commission of abuse’.^17^ In their scoping review, the authors contend that power is a central determinant of sexual violence in the context of the relationship between athletes and coaches. In this connection, they argue that the relationship of dominance and submissiveness between the coach and the athlete is derived from the ‘closeness of the relationship, the legitimate authority of the coach, the coach's expertise and previous successes, and the coach's ability to control access to the athletes.’^18^ This close relationship clouds athletes’ judgment such that they might not scrutinize the coach's behavior or recognize feelings of discomfort within the coach–athlete relationship because the coach makes use of his power in small steps. When these athletes finally realize the sinister nature of what is happening, they generally do not change sport club because they fear the negative consequences associated with the coaches' abuse of power or ‘authoritarian leadership’.^19^ Gaedicke et al.’s assessment of sexual violence in sport is shared by several other scholars.^20^

## The response of sporting federations

In addition to establishing Ethics Commissions,^21^ several of the major international sporting federations and national governing bodies have adopted Codes of Ethics which seek to prohibit sexual violence against athletes.^22^ By way of example, Articles 23 and 25, respectively, of FIFA’s Code of Ethics provide:

Article 23 Protection of physical and mental integrity

1. Persons bound by this Code shall protect, respect and safeguard the integrity and personal dignity of others.

2. Persons bound by this Code shall not use offensive gestures and language to insult someone in any way or to incite others to hatred or violence.

3. Harassment is forbidden.

Harassment is defined as systematic, hostile and repeated acts intended to isolate or ostracise or harm the dignity of a person.

4. Sexual harassment is forbidden.

5. Threats, the promise of advantages and coercion are particularly prohibited.

6. Violation of this article shall be sanctioned with an appropriate fine of at least CHF 10,000 as well as a ban on taking part in any football-related activity for a maximum of 2 years. In serious cases and/or in the case of repetition, a ban on taking part in any football-related activity may be pronounced for a maximum of five years.

**Article 25 Abuse of position**

1. Persons bound by this Code shall not abuse their position in any way, especially to take advantage of their position for private aims or gains.

2. Violation of this article shall be sanctioned with an appropriate fine of at least CHF 10,000 as well as a ban on taking part in any football-related activity for a minimum of two years. The sanction shall be increased accordingly where the person holds a high position in football, as well as in relation to the relevance and amount of the advantage received.

Meanwhile, Article 1 of the International Olympic Committee’s Code of Ethics provides that all persons formally connected with the Olympic movement must respect certain fundamental ethical principles, including:

1.4 Respect for international conventions on protecting human rights insofar as they apply to the Olympic Games’ activities and which ensure in particular:

- respect for human dignity;

- rejection of all forms of harassment and abuse, be it physical, professional or sexual, and any physical or mental injuries;

In similar vein, Article 2 of the International Tennis Federation’s Code of Ethics provides:

2.1. Officials must:

2.1.1. act in accordance with the highest standards of honesty and integrity in all of their activities as Officials;

2.1.2. respect human rights that may be impacted in their actions as Officials, including:

2.1.2.3. not committing any form of harassment or abuse of any person, whether physical, professional, sexual, psychological or otherwise.

Article 2 of the International Cricket Council’s Code of Ethics reads that ‘all forms of harassment (whether physical, verbal, mental, sexual or otherwise) are prohibited’, while Article 130 of the International Basketball Federation’s (FIBA) Internal Regulations provides that ‘all forms of harassment, vilification, and abuse by Basketball Parties, be it physical, professional or sexual, and inflicting, facilitating or tolerating any non-accidental physical or mental injuries are strictly prohibited.’

While it is certainly commendable that some governments and international federations, as described above, have adopted specific instruments that seek to militate against sexual violence committed in the context of sport, these rules do not and have not operated as a panacea. From a Feminist Perspective, the mere existence of these rules is not sufficient to combat sexual violence in sport; robust rules, backed by equally robust enforcement, are apposite.

## Feminist approaches to sexual violence

Feminism is a global movement that seeks to end, in the words of Bell Hooks, sexism, sexist exploitation and oppression.^23^ The movement is not new, nor has the progress made by Feminist scholars in bringing the lived experiences of women, according to Diane Gill, from the marginalized status (‘other’) to the center,^24^ been an easy battle. In fact, for many years, Feminist scholars have waged war against the historically inaccurate narratives that sexual violence is merely an act of sex, that women are complicit in their sexual exploitation, and that women are men’s property.

Several strands of Feminism attempt to explain sexual violence, within and outside of sport, against women and girls. The earliest of these approaches is that of Liberal Feminism, whose focus is on advancing policy and legal changes that would foster equal economic and social opportunity for women. Reflecting on Liberal Feminism in the context of sexual violence against women, Sascha Canan and Mark Levand^25^ posit that the approach is premised on the assumption that sexual violence can be resolved through structurally equal treatment of women and men. This approach has been criticized, however, for playing by the rules in seeking only to address legal and economic issues broadly, rather than the role of patriarchy as a social system in oppressing and subordinating women.

By contrast, Radical Feminism places considerable emphasis on patriarchy as the key cause of gender inequality and violence. By patriarchy, Canan and Levand refer to a social system that values traditional masculine social norms, whereby men are viewed as strong, stoic, powerful, and sexually aggressive, and where men occupy positions of power.^26^ Radical Feminists, like Susan Brownmiller, do not see sexual violence against women as a mere random act, but as a product of social domination by men.^27^ Brownmiller considers that while sexual violence is sexual, it is never ‘sexy’ because this type of violence is motivated by male domination and female degradation, often reflected in a conscious process of intimidation to keep women in fear.

Similar views have been articulated by leadings Feminist scholar Catharine MacKinnon,^28^ who argues that sexual violence is as much about power and control as it is about sex. She contends that sexual violence is not an isolated act or moral transgression, but rather an act of terrorism and torture against women. It involves, in her view, aggression against women who are perceived as having less power than their male counterparts.

Contemporary scholars like Beverly McPhail endorse the Radical Feminist view that sexual violence is a political matter, and not just a personal or isolated act.^29^ In her view, sexual violence operates as a means of enforcing gender roles in society and maintaining hierarchy in which men retain control. She, however, acknowledges that Radical Feminism does have its limits in that its near-ubiquitous focus on power and control, which does not fully account for the etiology of said violence, which might be rooted in male sexual aggression. She advocates instead for a Feminist Framework Plus, in which she argues for ‘theory-knitting’^30^ between the different strands of Feminism to account for the complex relations between men and women in society.

While Liberal and Radical Feminisms are, of course, the most well-known of the Feminist theories, there are other ground-breaking theories of Feminism which seek to problematize the commission of sexual violence against women. Intersectionality is one of these theories. Made popular by Kimberle Crenshaw in the 1980s,^31^ this theory posits that some women experience intersecting inequalities in society qualitatively different from their counterparts along the praxes of power, gender, race, class, nationality, and age which place them in an especially vulnerable position in so far as their experience of sexual violence is concerned. Crenshaw notes that women of colour have historically experienced the world in a qualitatively different manner than their white counterparts because their gender, race, and social class, from the time of slavery till now, have been appropriated to reinforce a sexual hierarchy in which they are not only devalued, but subject to racial and sexual subordination. In this regard, Crenshaw contends that black women are more likely to experience sexual violence than their white counterparts. Whereas Intersectionality is a broad analytical framework that assesses vulnerability to sexual violence across multiple praxes, including but not limited to social class, Marxist Feminism takes the approach that sexual violence can principally be explained by the lower social class of women in society, with gender disparities only considered a secondary site of vulnerability.^32^

In the final analysis, the combined effect of the different strands of Feminism described above is a movement that treats male sexual violence as perpetuating male dominance and patriarchy. The goal of Feminism, then, is to challenge the structural nature of patriarchy which, according to Louise Pedersen, results in women’s voices being unappreciated and unheard and their bodies exploited.

The ensuing cases, while dark and uncomfortable, are an attempt to bring to the fore the recent lived experiences of women who have suffered unimaginably at the hands of a sporting regime that is steeped in patriarchy.

### Karim Keramuddin v. Fédération Internationale de Football Association (FIFA)^33^

This was an appeal brought before the Court of Arbitration for Sport (CAS) by Keramuddin Karim, an Afghan national who was the President of the Afghanistan Football Federation (AFF) from 2004 until December 2018. He appealed against a decision of the Adjudicatory Chamber of the FIFA Ethics Committee, which held that he had violated Articles 23 (‘protection of physical and mental integrity’) and 25 (‘abuse of position’) of the FIFA Code of Ethics (FCE). He was subject to a lifetime ban from taking part in any football-related activity at the national and international level coupled with a fine of CHF 1,000,000. On appeal, he refuted allegations that he had sexually and physically abused players from the AFF women’s national team.

The CAS heard evidence from five players, although the ruling only addressed the allegations of four players—players A, B, C and D.

Player A recalled that Karim took her to a room in the new Afghan Football building, held her hand, pulled her towards him, and forced her to sit next to him. He then forcibly hugged her and tried to kiss her. When she cried and asked not to be molested, he insisted that she make no noise, and tried to hug and kiss her again, at which point he threw a piece of paper into her face after she again resisted. While attempting to open the door to leave the room, Karim shouted at her, indicating that from that time forward they were ‘no longer friends.’ Player A reported that, after the incident, she was negatively affected in myriad ways; she was expelled from the AFF after she complained about Karim's conduct; and suffered much vilification after Karim told her teammates that she was a lesbian.

Player B similarly reported that, on one occasion, when she visited a room in the new Afghan Football building, Karim approached her, forcibly took her hands, and asked that she have sex with him or at the very least kiss him. He promised player B that, were she to allow this, he would arrange for her to go on trips abroad with the national team, and that her salary would be increased. She refused his offer, but at great personal and professional loss. She reported that she was labelled a lesbian by Karim, and, having been prevented from engaging in football activities for the AFF, was not able to join domestic football clubs. What is also troubling about this ordeal was the fact that Karim reportedly said to her, 'I can touch you wherever I want to and you cannot stop me', which was not only inappropriate but highly misogynistic.

Meanwhile, Player C was a victim of rape by Karim in circumstances where she had asked him for a small amount of money to pay a taxi to take her home, as she did not have the requisite fare on the day in question. When she attempted to resist Karim’s forcible pursuit, he questioned whether she was a boy or girl, and later indicated to her colleagues that she was a lesbian. Of course, in a highly religious society as Afghanistan, Player C suffered not only the sexual assault, but the alienation, stigma, and discrimination that arose from the allegation that she was a lesbian coupled with the reality that she was no longer a virgin in a highly conservative society. Karim appeared to be not only calculated in dealing with Player C, but treated her with a high degree of callousness by punching her in the face, and in throwing three/four hundred dollars at her, and then kicking her out of his office, after the incident. Cumulatively, these infractions resulted in Player C becoming socially withdrawn, and even suicidal. She was also distraught by the fact that he threatened to shoot her if she reported the matter to the authorities, as she intimated that she would do.

Separately, Player D reported that she too was a victim of Karim's sexual assault. More particularly, she complained that Karim viciously tore her clothes off on one occasion, notwithstanding her plight to the contrary. After he eventually allowed her to leave the terribly unpleasant situation, he began making false allegations against her that she was a lesbian. He also orchestrated her being kicked out of the AFF, and threatened that he would put her and her family's life in danger were she to make the incident public.

Before the CAS, Karim was found to have breached Articles 23 and 25 of FIFA’s Code of Ethics. Regarding Article 23(1) FCE, the CAS found that Karim breached his duty to protect, respect and safeguard the integrity and personal dignity of the players of the AFF’s women’s national team. More particularly, the tribunal was unequivocal in its finding that, by verbally and sexually assaulting and abusing Players A, B, C and D and by raping Player C, Karim failed to uphold the principles of protection and safety embedded in Article 23(1) FCE. Indeed, instead of providing a safe environment for the players at the AFF, over which he presided as the highest-ranking official, and protecting their physical and mental integrity, ‘he created a violent, dangerous and horrifying atmosphere for them and committed despicable and heinous acts against them.’^34^

Turning its attention to Article 23(4) FCE, the CAS found that Mr. Karim engaged in sexual harassment of the players in question as result of his unwelcome and highly offensive sexual advances that were not solicited or invited. Having regard to test of whether a reasonable person would regard his impugned conduct as undesirable or offensive,^35^ the CAS did not hesitate to find that Karim sexually harassed, both verbally and/or physically, Players A, B, C and D. According to the CAS, the evidence clearly established that Karim inappropriately spoke to the players, sexually assaulted them, and even raped Player C.

The CAS then considered whether Article 23(5) FCE was breached. In this connection, the CAS found that Karim had not complied with his duty to refrain from threating, coercing or making promises of advantages to the players under his supervision. The CAS found evidence that Karim pointed a gun at Player C after having raped her, threatening to fatally shoot her in the head if she did not keep quiet about the incident. Additionally, the CAS held that Karim violated Article 23(5) FCE by offering Player B sporting and financial rewards to induce her to acquiesce to his sexual advances and threatened to fire her if she did not.

With respect to Article 25 FCE, the CAS considered that, for 5 consecutive years, between the years 2013 and 2018, during which he served as the AFF President, Karim, on AFF premises, sexually harassed and abused Players A, B, C and D and even raped Player C, all of whom had gone to see him in his capacity as AFF President and for official business. The CAS Panel condemned the fact that, in this connection, Karim had misused his position as the AFF President and, more specifically, the players’ sporting and financial dependency on him for private aims and gains—i.e., to try to obtain sexual favors (e.g., Player B to whom he offered a spot on the national team and a salary raise in exchange thereof) or simply to get the players to meet him personally in his offices so that he would be in the position to sexually harass and abuse them (e.g., Player A who went to collect a letter as instructed, Player C who went to obtain money owed, and Player D who followed the Karim’s instructions and went in the belief that she would have a meeting about her absences and potential expulsion). In this light, the CAS did not hesitate to find that Karim took advantage of his position as the AFF President for private aims or gains and thus violated Articles 25 FCE.

Having regard to the foregoing, the CAS upheld the Adjudicatory Chamber of the FIFA Ethics Committee’s sanction of a lifetime ban from taking part in any football-related activity at the national and international level and a fine of CHF 1,000,000 for his violations of Articles 23 and 25 FCE. The CAS, in finding that the sanction in question was proportionate, considered the nature of the offence; the substantial interest in deterring similar misconduct; the offender’s assistance to and cooperation with the Ethics Committee; the motive; the circumstances; the degree of the offender’s guilt; the extent to which the offender accepts responsibility, and whether the accused mitigated his guilt by returning the advantage received, where applicable.^36^

On the question of the nature of the infractions in question, the CAS considered that Karim’s conduct was ‘extremely serious’ and ‘of the most serious, illegal, and immoral kind’,^37^ particularly having regard to the fact that he abused his position as the AFF President. In the Panel’s view, the violation of the players’ integrity and personal dignity, the sexual harassment, and the threats and promises of advantages were only possible due to his position as the highest-ranking official of the AFF which necessitated the imposition of the maximum sanction allowable under the FIFA regulations – a lifetime ban and a fine of CHF 1,000,000. The CAS accepted that the imposition of this sanction was necessary not only because of the nature of the infractions in question, but also because of the ‘obvious and substantial need’^38^ to deter similar misconduct in the future from Karim, as well as from any other FIFA officials. In its landmark dicta that merits articulation here, the CAS noted that:

It is an offence that is disgraceful and which cannot be tolerated from any official, let alone the president of a national federation, who bears the responsibility to set a proper example for the federation’s employees, the others individuals affiliated thereto, and more generally to all those involved in the world of football.^39^

The CAS’s profoundly insightful dicta did not end there; it went on to express that ‘this is a case of unprecedented gravity’.^40^ In this regard, it rejected the view that corruption related offenses were as serious as the sexual violence committed in this case. More pointedly, it noted that:

[the] offenses of bribery, corruption and match-fixing, while very serious in their own right, are far less severe than the vile and horrendous offenses committed by the Appellant which have never before been (and hopefully never again will have to be) dealt with by the CAS. Indeed, unlike bribery and match-fixing which damages the integrity of the sport, the offenses committed by the Appellant violated basic human rights and damaged the mental and physical dignity and integrity of young female players. With his appalling acts, the Appellant has destroyed not only their careers, but severely or irreparably damaged their lives. As such, they warrant the most severe sanction possible available under the FCE.^41^

Having regard to the foregoing, the CAS Panel confirmed that the life ban and CHF 1,000,000 fine was proportionate to the violations committed.

The different, but mutually reinforcing, strands of Feminism described earlier in this Article can usefully contextualize Karim’s malevolent treatment of the four young female Afghan footballers in this case. From a Liberal Feminist perspective, Karim’s depraved conduct highlights the glaring economic and social disparities in Afghan football, which are reflective of wider societal disparities, and which created an enabling environment for Karim, a powerful man, to unleash unmatched sexual violence against those whom he perceived to be less powerful. Meanwhile, Marxist Feminists would argue that the primary basis undergirding the sexual violence meted out to the female players was class inequality, with their gender being only a secondary concern. More particularly, Marxist Feminists would contend that Karim was only able to rape Player C because of her lower social class; that is, she had no money, and therefore had to ask him for money to pay a taxi to take her home. Her vulnerability in this regard was used by Karim to justify her exploitation. Similarly, in respect of Player B, Karim was able to use her lower social class as a basis for demanding sexual favours, while promising to arrange for her to go on trips abroad with the national team, and an increase in her salary.

Neither Liberal nor Marxist Feminism, however, provides a fulsome analysis of the complexities evident in this case. In this regard, the approach of Radical Feminists must be countenanced in this analysis. From a Radical Feminist perspective, Karim’s sexist, violent and oppressive behaviour is one of the clearest manifestations of patriarchy. More pointedly, Susan Brownmiller would not see the sexual violence meted out against the women in this case as a mere random act, but as a product of patriarchy—social domination by men and commensurate female degradation. Brownmiller would interpret several aspects of Karim’s conduct as acts of intimidation aimed at creating fear in the Players, and which in turn reinforced Karim’s powerful status and domination, namely; throwing a piece of paper into Player A’s face; telling Player B that he could ‘touch [her] wherever [he] want[ed] to and [that she] cannot stop [him]'; punching Player C in the face; throwing three/four hundred dollars at her; and making false allegations against Player D that she was a lesbian and threatening that he would put her and her family's lives in danger.

Similarly, Catharine MacKinnon would interpret Karim’s conduct as much about power and control as it was about sex. For her, Karim’s conduct would not be characterized as an isolated act or moral transgression, but as systematic acts of terrorism and torture against the female Players. Because the Players were perceived as having less power than him, Karim used his social influence and position of power to not only sexually abuse the Players, but expel them from the Federation, and thus ruin their chances of ever progressing professionally. In the words of Beverly McPhail, we ought not to simply conceive of Karim’s violence as a personal act against the Players; his conduct was a political statement against vulnerable women that served to reinforce harmful gender roles while further embedding a hierarchical structure where men are in control.

The Intersectional Approach could also explain the sexual violence meted out against the Players. In keeping with Kimberle Crenshaw’s analysis, these women—because of their intersecting axes of vulnerability—their gender, age, social class, race, and religion—were especially vulnerable to Karim’s sexual violence. More particularly, Karim used the Players’ financial disenfranchisement and their relatively youthful age, as well as the strict religiosity of Afghanistan, now ruled by the Taliban, to not only commit depraved acts, but justify his behaviour. Indeed, on several occasions, Karim sought to describe the women as homosexuals when they attempted to resist his sexual advances, knowing fully well that in a strict religious State as Afghanistan not only will these allegations serve to legitimize his behaviour, but create further oppressive professional barriers for the affected Players. In this regard, Karim’s conduct, which was steeped in patriarchy, was not merely acts of sexual subordination, but systematic acts aimed at devaluing the women concerned. Through his sexual violence and religious bigotry, Karim sought to make, and succeeded in making, the women feel worthless and even suicidal, and through subsequently expelling them from the Federation, he robbed them of their hopes and dreams in the most cataclysmic fashion.

### Yves Jean-Bart, Decision of the Adjudicatory Chamber of the Ethics Committee (18 November 2020)

This case concerned Yves Jean-Bart, a Haitian national, who was, prior to the hearing of this case, the President of the Fédération Haïtienne de Football (FHF) since 2000. In addition, Jean-Bart was also a member of the Committee for Women's Football and of the FIFA Women's World Cup (27 September 2002 -11 November 2005; a member of the Member Associations Committee (2 December 2005—2 January 2012); and a member of the Organizing Committee for the FIFA Confederations Cup (3 January 2012—18 January 2017). After several serious allegations of systemic rapes and other acts of sexual violence within the FHF became public, a hearing by FIFA’s Investigatory Chamber was held. The Chamber imposed a provisional ban of 90 days on Jean-Bart from taking part in all football-related activities at the national and international level, in accordance with Article 84 of the FCE 2019. Jean-Bart appealed the case to the Adjudicatory Chamber of FIFA’s Ethics Committee.

Victim A testified that, during an Under-17 competition, Jean-Bart invited her to see him at his hotel room. When she arrived, he presented her with a pack of underwear. He then pulled her toward him in an attempt to sexually assault her, but she resisted. This catalyzed a series of adverse consequences, including intimidation and retention of her passport which prevented her from applying for a student visa, culminating in her losing a university scholarship.

Meanwhile, Victim B reported that while a member of the U-17 team, Jean-Bart invited her to his room, at which point he handed her a bag of underwear. He then pulled her close to him and fondled her. Being disturbed by the infraction, she pushed him away and fled. She also reported that, on a separate occasion, when travelling in a car with Jean-Bart, he touched her inappropriately for the duration of the trip, although she tried to repel his advances. He later threatened that, because of her resistance to his advances, he would never assist her in her professional endeavours. She was also prevented from pursuing further studies abroad because Jean-Bart refused to give back her passport. In fact, Jean-Bart indicated that he would not give back her passport unless and until she formed a relationship with him.

Separately, other sources (including The Fédération Internationale des Associations de Footballeurs Professionnels (FIFPro) and Human Rights Watch (HRW)), reported several instances in which Jean-Bart used promises of contracts or scholarships and the threat of expulsion from the National Training Centre as a means of pressuring the young female players to have sex with him. Another reported instance of this type of appalling conduct allegedly occurred when, at only 14 years old, a young female footballer was violently raped by Jean-Bart at the Ranch in Haiti. This player was reportedly kept from seeing her family for the summer holidays after she became pregnant. She was then reportedly taken by a FHF official to have an abortion when she was five months pregnant.

One of the witnesses who testified at the hearing also pointed to cases in which Jean-Bart took female players, including those who were not particularly skilled, abroad, and forced them to miss training sessions so that he could sleep with them. It appears that Jean-Bart was particularly successful in this respect because of his ability to refuse to give his victims their passports should they reject his advances. Another witness pointed to an occasion in which a player had gone missing after she had been subject to sexual violence at the hands of Jean-Bart. When she eventually emerged, a medical examination was conducted; the medical certificate showed very clearly that she was exposed to sexual assault at the hands of Jean-Bart. Yet, still, another player was reported by a witness at the hearing as having left the Centre before the COVID-19 lockdown after she discovered she was pregnant for Jean-Bart.

Interestingly, the witnesses also testified that even after Jean-Bart was provisionally suspended, he still visited the Centre and other FHF facilities, giving away money and intimating that he would soon be back. This was apparently aimed at intimidating his victims and the staff of FHF from giving evidence. Interestingly, Jean-Bart, after his provisional suspension, also surreptitiously arranged with one of his fellow members of staff to extract several passports belonging to young female players from the FHF office.

Before the Adjudicatory Chamber of FIFA’s Ethics Committee, violations of Article 23 FCE (protection of physical and mental integrity) and Article 25 (abuse of position) were alleged. In respect of the former, the Adjudicatory Chamber considered that, although the FHF should have been a safe environment for all players, Jean-Bart's conduct created an environment of ‘fear, danger and frustration for the affected players at the Centre.’^42^ The Adjudicatory Chamber recalled the evidence of Victim A who spoke of having been intimidated and alienated by Jean-Bart and being unable to apply for a student visa because of his retention of her passport. She also testified of living in fear after having resisted Jean-Bart's advances, since he, at one point, indicated to her in a threatening manner that he knew where to find her. Similarly, Victim B was prevented from pursuing her university programme on account of the retention of her passport by Jean-Bart. In this connection, the Adjudicatory Chamber considered that:

These two painful testimonies paint a picture and a pattern that has been confirmed by other witnesses and sources, of the Centre as a place where girls of poor social background would come to in search of sportive and financial success or fame, only to wake up to the brutal reality of sexual violence, living in constant stress and fear, discovering that a refusal of Mr. Jean-Bart’s indecent proposals equaled failure.^43^

The Adjudicatory Chamber also found Jean-Bart to have systematically and repeatedly committed acts of persecution against the victims/witnesses, as well as having threatened their relatives, vandalized their homes, and offered money with the intention to intimidate and prevent these victims/witnesses from testifying.

In terms of sexual violence, harassment and exploitation, the Adjudicatory Chamber was emphatic in its condemnation of Jean-Bart's visits to the Centre and frequent travels in the company of minors (to the doctor and elsewhere); sexual encounters at the Centre; the rape of a minor and abortion of the unwanted pregnancy the kidnaping of a girl; as well as a ‘competition’ among senior officials of the FHF where the goal was to rape as many girls and/or take as many girls’ virginity as possible. The Adjudicatory Chamber described these infractions as 'appalling behavior which occurred over a longer period of time, and which was part of a system of sexual violence and exploitation of (minor) girls.'^44^

In so far as the threats, promise of advantages and coercion were concerned, the Adjudicatory Chamber condemned the fact that the sexual advances of Jean-Bart towards the (minor) female players were always based on the influence (threats or coercion, or by the offering of gifts and the promise of various benefits) that the latter wielded over the girls. In this connection, the Adjudicatory Chamber considered that the threats, coercion and promise/offering of benefits by Jean-Bart to exert his reign of sexual violence over the female players at the Centre was 'a despicable conduct that was not only intentional, but also premeditated and methodical.'^45^ In short, his conduct amounted to a serious breach of Article 23 FCE.

Separately, in relation to Article 25 FCE, the Adjudicatory Chamber found that Mr. Jean-Bart had abused his position as President of the FHF. Among other things, the court recalled the evidence of Victim A who testified of having been intimidated, alienated, and refused a professional contract and the opportunity to further studies after she rejected Jean-Bart's advances. The Adjudicatory Chamber also held that Jean-Bart found other ways in which he could affect the young players’ careers or sportive situation by abusing his authority as FHF president, namely: by expelling players from the Centre; approving the list of players that would be part of the national team and who would receive visas to participate in football matches/competitions abroad; imposing them in a team despite injury or their performance being sub-par; and detaining the players’ passports in the FHF offices and refusing to return them to the players when requested to do so. Moreover, all the players at the Centre were closely scrutinized and measures were taken to prevent anyone from reporting or disclosing the abuse to outsiders. Among other things, Jean-Bart, even after he was provisionally suspended, still visited the Centre and other FHF’s facilities and, during these visits, he offered money as a bribe so as to convince and assure victims and staff within the FHF that he would be shortly back in charge of the federation, thus discouraging them from testifying.

In the final analysis, having regard to the foregoing, the Adjudicatory Chamber determined that:

Mr. Jean-Bart abused of his position as the most senior official in Haitian football, as president of the FHF (for 20 years) and created a very complex and extremely harmful system of sexual violence and exploitation of female players, also minors of age, which occurred inside and outside of the Centre and shattered the lives and careers of young girls coming from vulnerable backgrounds with their passion of playing football and possibly pursuing a football career. By doing this, Mr. Jean-Bart also betrayed Haitian football as well as the fundamental values of the Game and of FIFA. He became an absolute ruler of an organization, irreversibly corrupting his presidential mandate as custodian of the football youth in his country.^46^

It further noted that:

Mr. Jean-Bart’s behaviour is simply inexcusable, a disgrace for any football official. The pain and suffering he has caused his various victims of sexual harassment and abuse cannot even be fully comprehended, and represents a very dark stain on the image and reputation of football as a sport loved by so many, whose principal value and credo is “fair play”. While claiming that he was developing Haitian football, in particular women’s competitions and teams, Mr. Jean-Bart did the exact opposite: he abused his position in order to satisfy his personal attitude of domination over the most fragile people, destroying the careers and lives of young promising female players. In addition, no acts of mere negligence are at stake here but deliberate actions.^47^

In imposing a worldwide lifetime ban on Jean-Bart and a fine of CHF 1,000,000, the Adjudicatory Chamber usefully opined that:

(...) conduct such as that of Mr. Jean-Bart affects the very core of sports and is nothing less than life threatening for sports and sports organisations. Thus, if officials who are found guilty of sexual harassment and assault, committed through a clear and systematic abuse of position, remained within the sports structures, this would cause irreparable damage to sports and football in general and to the FHF, CONCACAF and FIFA, in particular. In cases like the present one, the only means to save sports from enormous reputational damage is a determined and resolute sanctioning of the persons concerned.^48^

From a Radical Feminist perspective, Jean-Bart’s conduct is yet again another clear illustration of how patriarchy operates to reinforce gender inequality and ingrain institutionalized violence against women. Jean-Bart, an obviously powerful man, was able to use that power and influence to create a shockingly hostile environment for the female players whose worth was equated to packs of underwear. To use the words of Susan Brownmiller, Jean-Bart’s reprehensible conduct must not be merely viewed as an isolated random act, but rather part of a systematic manifestation of social domination by men. This system of social domination enabled Jean-Bart to not only sexually exploit these vulnerable players over many years, but also withhold from them even their government issued identity documents, and, by extension, scholarship opportunities to further their professional development. For Brownmiller, the violent sexual acts committed against the players, coupled with the many threats that followed, served only to intimidate the players and thus keep them in fear that their lives and those of their families may be in danger if they were to report the matter. While disconcerting, this is not atypical of a system that is steeped in patriarchy.

Meanwhile, from the perspective of Catharine MacKinnon, Jean-Bart’s conduct would be viewed as much as about power and control as it was about sex. Sexual violence, for Jean-Bart’s, was a way of terrorizing and torturing the players, while demonstrating his toxic masculinity in a society that values male sexual aggression. That his reach extended to getting the players pregnant, arranging abortions, and getting the assistance of other members of his staff to keep the players in check, even after he was suspended, learly illustrate that patriarchy is alive and well, even in the sporting world. Indeed, from an Intersectionality perspective, the players’ gender, class and age operated, to use the words of Kimberle Crenshaw, as sites of exploitation whereby Jean-Bart could use his position as a powerful, older man to commit grave acts of sexual violence. The treatment of the players’ bodies as mere objects of gratification, while not an isolated act in a society built on patriarchy, once again reinforces why Feminist perspectives are important in bringing, in the words of Diane Gill, ‘women's experiences from marginalized status (or "otherness") to the center.’^49^

## A Sobering Reality

The foregoing cases tell a frightening story of vulnerability, objectification, exploitation, and intimidation, which seemingly run deep, at least in the context of football, in some jurisdictions. That talented young women could be systematically subject to undignified sexual advances for several years without appropriate legal recourse ever becoming available until only recently is both mindboggling and symptomatic of a wider accountability problem in sport. Further, that a culture of impunity exists in the sporting realm in modern democratic societies that are supposedly built upon the universality of human rights and the rule of law is cause for our collective abhorrence.

The *Karim* decision is an important call to action for all concerned in the management and administration of football, and sport, more generally, as it raises searching questions regarding abuse of power, the exploitation of vulnerability, and the systematic failure of sporting codes of ethics to deter persons in positions of influence from exploiting others. That the press, and not internal disciplinary mechanisms, brought to the fore the grave indignity suffered by the girls in question sends a deeply disturbing message that in some jurisdictions sport is not a safe space for the development of talent. The fact that Karim was able to weaponize the girls’ resistance to his sexual advances by simply characterizing them as ‘lesbians’ is further evidence that sport, and, football, more specifically, may not be as safe a space as one would imagine.

Karim’s actions were not only patriarchal and misogynistic, but criminal. To have used his position of influence to exploit young, vulnerable girls from poor backgrounds over the course of many years merits not only sporting sanctions, but the imposition of criminal sanctions, akin to those imposed on Larry Nassar. More than this, however, given how easy it was for Karim to have perpetrated the acts in question over many years, the question invariably arises as to whether cases of this nature might only represent the tip of the iceberg. Whether or not this is the extant situation, it is evident that more robust governance and disciplinary arrangements need to be put in place to protect vulnerable players from exploitation. More pointedly, appropriate procedures against retaliation must be adopted to incentivize victims of sexual violence to come forward at the earliest opportunity to report the actions of sexual predators.

Notwithstanding the foregoing, if there is anything positive to take away from the *Karim* decision it would likely be the CAS’s conscientious treatment of the witnesses who appeared at the hearing. Despite resistance from Karim, the CAS granted an anonymity order, thereby enabling the witnesses to testify via an interpreter and audio-visual technology (namely, a voice scrambler). Although the CAS acknowledged that the right to a fair trial is recognised by Article 6 of the European Convention on Human Rights (ECHR) and Article 29(1) of the Swiss Constitution, it nevertheless considered that witness anonymity safeguards utilized did not, in this case, infringe upon Karim’s right to a fair trial. In this connection, the CAS recalled its earlier cases of *Contador*^50^ and *Pobeda*^51^ in which it outlined strict conditions for invoking witness anonymity safeguards, namely that the:

(i) witnesses motivate their request to remain anonymous in a convincing manner;
(ii) court has the possibility to see the witnesses;
(iii) witnesses would concretely face a risk of retaliation by the party they are testifying against if their identities were known;
(iv) witnesses are questioned by the court itself, which must check their identities and the reliability of their statements; and
(v) witnesses are cross-examined through an ‘audiovisual protection system’.

Having regard to the fact that the witnesses in this case were heard through ‘audiovisual protection’ and an in-depth check of their identity and reputation was conducted, the CAS considered that an adequate balance was struck between the procedural rights of Karim, on the one hand, and the necessity to protect the life and personal safety of the witnesses, on the other. Even further, the safeguards invoked by the CAS were deemed to be necessary to protect the witnesses against existing and/or future threats that had been made by a man with significant political influence and power in Afghanistan, who had threatened the lives of at least two players directly, even at gun point, and who was still at large, notwithstanding an arrest warrant that was out for him. In this connection, the CAS considered that disclosing the witnesses’ identities would create a serious potential threat to their lives and personal safety.

Beyond the *Karim* decision, the *Jean-Bart* case also raises significant questions about the vulnerability of women and girls to sexual violence in sport. As the facts of this case illustrate, Jean-Bart and his colleagues, Yvette Félix^52^ and Nella Joseph,^53^ were able to use the institution of sport to perpetrate gross violations of the players’ human rights for many years, while the survivors were systematically silenced by a culture of fear, intimidation and, quite interestingly, the retention of their passports. The egregious nature of Jean-Bart and his colleagues’ conduct cannot be overstated; the abuse of power, impunity, and systematic reprisals should agitate everyone who is genuinely concerned with creating a safe sporting environment for women and girls, in particular. How can women and girls be expected to demonstrate athletic prowess when the environment within which they are forced to live, study, and hone their sporting skills is antithetical to basic tenets of human decency?

That Jean-Bart and his colleagues were allowed to engage in a most pernicious activity that compromised the lives and livelihoods and inherent dignity of the players in question should rightly spark public outrage. While FIFA’s Codes of Ethics exist, those charged with monitoring, supervision and its overall implementation clearly did not demonstrate the type of due diligence that was required by the relevant circumstances of these cases. In addition, that the Confederation of North, Central American and Caribbean Association Football (CONCACAF), when contacted by FIFA about the allegations, indicated that it did not have an established Ethics Committee at confederation level, and therefore, it was unable to properly investigate the case further is also indicative of a systematic failure of the institution of sport to adequately protect the interests of the girls in question from exploitation. In the same vein, that the Haitian government, having heard the allegations of sexual violence over the years, did not act immediately to circumscribe the surreptitious activities of Jean-Bart and his associates is frightening and frankly illustrative of a state where human rights and the rule of law are second rate principles.

## A Call to Action

While sporting bodies can adopt Codes of Ethics and establish Ethics Commissions for the purpose of investigating allegations of sexual violence, these alone will never be enough given the jurisdictional limitations of sporting bodies. In this regard, sporting bodies, along with national governments, must work synergistically to combat the scourge of sexual violence against women and girls in sport.

### The Role of States

States have a constitutional duty to protect persons within their jurisdiction, including athletes, from sexual violence. In practice, this duty of protection requires the application of the criminal law to penalize those who engage in sexual violence, as well as the application of human rights law to assist and support athletes who are subject to sexual violence. Outlined below are several of the practical ways in which States could effectively respond to sexual violence in sport:

- commission regular quantitative and qualitative research on the status of sexual violence in sport. This research should, at a minimum, identify the socio-cultural issues that enable sexual violence in sport, the profile and modus operandi of perpetrators, and profile and needs of victims/survivors;
- enact legislation, if there are none, to require that assessment of the criminal history of paid and volunteer staff who interact with children and young people, the prohibition on those convicted of and those subjected to disciplinary sanction from performing roles involving regular contact with children, and the requirement that everyone must report violence committed against athletes, especially children and young people, to the police and to an independent contact point in sport, if any.
- perpetrators should be robustly prosecuted and convicted. Convicted offenders and those subjected to disciplinary sanctions should be prevented from playing an active role in sport. Perpetrators with a history of sexual offences who are prevented from performing roles in a sporting environment should be entered in a Criminal Records Information System. These individuals should be prohibited from working in a paid or volunteer capacity within sport;
- raise awareness about sexual violence in sport, in conjunction with sporting bodies and non-governmental actors. These activities should encourage those affected to report incidents of sexual violence to the police and to an independent contact point in sport, if any. This will help to address the problem of underreporting. Awareness-raising programmes should also highlight the impact of sexual violence on athletes’ welfare and performance, making it clear that this type of behaviour in sport is prosecutable by law, and urging the public to report incidences of gender-based violence in sport.
- sporting bodies should be mandated to implement procedures that would identify parties responsible for the commission of sexual violence and provide systems to support those adversely affected by sexual violence. This might, for example, entail the adoption of standard grievance and protection procedures (including complaint and reporting procedures, and the creation of an independent contact point) to handle reports of sexual violence in sport;
- develop a coherent national policy framework (i.e., a national strategy) to fight sexual violence in sport. This framework should explicitly acknowledge sport as a setting where sexual violence occurs. It should also identify the legal and policy initiatives in place to combat sexual violence in sport;
- an inter-sectorial group (comprised of policy officers from relevant sectors and representatives of sport governing bodies and civil society organisations) should be convened which, under the direction of the relevant National Sports Council, should coordinate the operationalization of the national policy framework; and
- services to support those subject to sexual violence in sport, whether run by or independently of sport, should be provided.

### The Role of Sporting Federations

Notwithstanding their inherent jurisdictional limitations, Sporting Federations also have an important role to play in the eradication of sexual violence in sport. Outlined below are several of the practical ways, endorsed by the European Commission, in which Sporting Federations could effectively address sexual violence in sport:

- establish an independent contact point (also known as ‘Trust Person’ in some countries) so that those affected by sexual violence in sport have a named person to whom they can disclose violations. This contact point should have operational responsibility for managing reports of sexual violence, embedding relevant policies and procedures, and championing sexual violence prevention initiatives. The independent contact point should be provided with sufficient support (training and resources) to effectively carry out this role. The existence of this independent contact point needs to be made known within the sporting community. Anonymous reporting mechanisms should also be considered;
- given the intense and close relationship that coaches tend to have with athletes under their supervision, they must be fully sensitized about the dangers of blurring the lines of acceptable conduct. They should also be sensitized about signs to look out for in respect of those who might be subject to acts of sexual violence from sport staff or peers. They should be bound to report such situations;
- national sporting bodies that do not comply with minimum standards, including procedures on reporting sexual violence committed against athletes, should be subject to sanctions by international sporting federations, including suspension and funding cuts;
- develop minimum standards and templates to support the recruitment of staff and volunteers who demonstrate integrity in their dealings. These standards could be included in a Code of Ethics and Conduct that sport staff are required to commit to when undertaking any paid or volunteer work in the sports organisation;
- develop rules and regulations, based on known risk factors, to protect athletes. These can be referred to in a Code of Ethics and Conduct. Examples may include banning coaches from being left alone with athletes, or sleeping in the same hotel room as athletes, or entering the locker rooms alone and unannounced, or engaging in physical contact with athletes’ bodies without their (and where the athletes are children, their parents’) consent;
- develop mandatory sport-specific training and education modules on risk assessment and prevention and intervention approaches to raise awareness about sexual violence in sport. Tailored training should address all relevant actors (including sport policymakers, club management, coaches, sport medical and support staff, and those in related roles, as well as athletes). Young athletes should also be sensitized to the issue and to reporting mechanisms;
- establish﻿﻿﻿﻿ stringent disciplinary procedures and sanctions to militate against retaliation against athletes and other persons who, in good faith, report incidents of﻿﻿﻿﻿ sexual violence; and
- undertake rigorous and independent research into incidents of sexual violence.

## Conclusion

This article has addressed one of the thorniest, but nevertheless most important, issues in sports law today—sexual violence committed against athletes in the sporting context. It has advanced the argument, from a Feminist Perspective, that, having regard to several recently decided cases, it can properly be said that the time is ripe for a reimagining of how sporting bodies and States address sexual violence perpetrated against athletes. While the article acknowledges that sport, being a microcosm of society, is not immune to social ills such as sexual violence, it nevertheless contends that the lived experiences of the countless victims who have suffered at the hands of sporting officials strongly suggest that extant Codes of Ethics and rules of procedure are not enough. A fundamental change in the way in which sport is administered and practiced is necessary to prevent vulnerable athletes from becoming victims of sexual violence, and to afford them appropriate legal redress and protection from retaliation in circumstances where they report acts of sexual violence committed against them.

## Footnotes

1 Readers may be interested in viewing the documentary on the Larry Nassar saga here: ‘At the Heart of Gold Inside the USA Gymnastics Scandal’ (HBO, 30 May 2019) https://www.youtube.com/watch?v=_w4_BOLsjF8

2 Jamie Abrams and Amanda Potts, ‘The Language of Harm: What the Nassar Victim Impact Statements Reveal About Abuse and Accountability’ (2020) 82 University of Pittsburg Law Review 71

3 Julie Novkov, ‘Law, policy, and sexual violence in the# MeToo movement: USA gymnastics and the agency of minor athletes’ (2019) 40(1) Journal of Women, Politics & Policy 42.

4 Anne Marie Burke, ‘Raising the Bar: Increasing Protection for Athletes in the Olympic Movement from Sexual Harassment and Abuse’ (2021) 31(1) Journal of Legal Aspects of Sport 60, 75.

5 Marc Edelman and Jennifer Pacella, ‘Vaulted into Victims: Preventing Further Sexual violence in US Olympic Sports Through Unionization and Improved Governance’ (2019) 61 Arizona Law Review 463.

6 Heather Udowitch, ‘The Larry Nassar Nightmare: Athletic Organizational Failures to Address Sexual Assault Allegations and a Call for Corrective Action’ (2020) 16 DePaul Journal of Sports Law 1

7 Emily Monnett, ‘Is Silence for Sale? The First Amendment Implications of the Michigan State University's Settlement with Larry Nassar Survivors’ (2019) 18 First Amendment Law Review 142.

8 Juan Martinez, ‘Sexual violence and Bankruptcy: How Organizations Abuse Chapter 11 to Avoid Victims' Demands for Answers’ (2020) 37(1) Emory Bankruptcy Developments Journal 213; Sarah Hartley, ‘USA Gymnastics: How Bankruptcy Proceedings Can Help Resolve Mass Sexual violence Claims’ *LawinSport.com* (21 June 2021) https://www.lawinsport.com/topics/item/usa-gymnastics-how-bankruptcy-proceedings-can-help-resolve-mass-sexual-abuse-claims

9 Jerry Moran & Senator Richard Blumentha, 'The Courage of Survivors: A Call to Action' (Senate Olympics Investigation, July 30, 2019) https://www.moran.senate.gov/public/_cache/files/c/2/c232725e-b717-4ec8-913e-845ffe0837e6/FCC5DFDE2005A2EACF5A9A25FF76D538.2019.07.30-the-courage-of-survivors--a-call-to-action-olympics-investigation-report-final.pdf

10 Jason Haynes and J. Tyrone Marcus, *Commonwealth Caribbean Sports Law* (Routledge 2019) chapter 8.

11 Anne Whyte, ‘The Whyte Review’ (Sport England and UK Sport, June 2020) 98

12 ‘Study on Gender-Based Violence in Sport’ (European Commission, European Union, 2016)

13 Angela Charlton, ‘Hundreds of French sports figures accused of sexual violence’ *AP NEWS* (April 3, 2021) https://apnews.com/article/sports-violence-sexual-assault-violence-in-sports-9e7459499b952a01be97eedb2dff88ea

14 Anne Marie Burke, ‘Raising the Bar: Increasing Protection for Athletes in the Olympic Movement from Sexual Harassment and Abuse’ (2021) 31 Journal of Legal Aspects Sport 60.

15 Jeannine Ohlert Jeannine, Ohlert Corinna Seidler, Corinna Seidler, Thea Rau and Marc Allroggen, ‘Sexual Violence in Organized Sport in Germany’ (2018) 48(1) Sportwissenschaft 59-68

16 Saul Marks, Margo Mountjoy and Madalyn Marcus, ‘Sexual Harassment and Abuse n Sport: The Role of The Team Doctor’ (2012) 46(13) British Journal of Sports Medicine 905

17 Sonja Gaedicke, Alina Schäfer, Brit Hoffmann, Jeannine Ohlert, Marc Allroggen, Ilse Hartmann-Tews, and Bettina Rulofs, ‘Sexual Violence and The Coach–Athlete Relationship—A Scoping Review from Sport Sociological and Sport Psychological Perspectives’ (2021) 3 Frontiers in Sports and Active Living 643707.

18 Ibid 8

19 Ibid

20 Marianne Cense and Celia Brackenridge, ‘Temporal and Developmental Risk Factors for Sexual Harassment and Abuse in Sport’ (2001) 7(1) European Physical Education Review 61; Ashley Stirling and Gretchen Kerr, ‘Abused Athletes' Perceptions of The Coach-Athlete Relationship’ (2009) 12(2) Sport in Society 227; Ingunn Bjørnseth and Attila Szabo, ‘Sexual Violence Against Children in Sports and Exercise: A Systematic Literature Review’ (2018) 27(4) Journal of Child Sexual Abuse 365; Kristine Bisgaard and Jan Toftegaard Støckel, ‘Athlete Narratives of Sexual Harassment and Abuse in the Field of Sport’ (2019) 13(2) Journal of Clinical Sport Psychology 226; Kari Fasting, Celia Brackenridge and Jorunn Sundgot-Borgen, ‘Prevalence of Sexual Harassment among Norwegian Female Elite Athletes in relation to Sport Type’ (2004) 39(4) International Review For The Sociology of Sport 373.

21 See, for example, Jason Haynes, ‘Commonwealth Games Federation launches new Ethics Commission’ (2022) 13(1) Sports Law and Taxation 16

22 Sylvie Parent and Guylaine Demers, ‘Sexual Violence in Sport: A Model to Prevent and Protect Athletes’ (2011) 20(2) Child Abuse Review 120.

23 Bell Hooks, *Feminist Theory: From Margin to Center* (Pluto Press 2000).

24 Diane Gill, ‘A Feminist Perspective on Sport Psychology Practice’ (1994) 8 The Sport Psychologist 411

25 Sasha Canan and Mark Levand, ‘A Feminist Perspective on Sexual Assault’ in *Handbook of Sexual Assault and Sexual Assault Prevention* (Springer 2019) 3.

26 Ibid 7

27 Susan Brownmiller, *Against Our Will: Men, Women and Rape* (Ballantine Books 1975)

28 Catharine Mackinnon, ‘Rape: On Coercion and Consent’ (1997) *Writing on The Body: Female Embodiment and Feminist Theory* 42.

29 Beverly McPhail, ‘Feminist Framework Plus: Knitting Feminist Theories of Rape Etiology into A Comprehensive Model’ (2016) 17(3) Trauma, Violence, & Abuse 314.

30 Ibid

31 Kimberlé Crenshaw, ‘Demarginalizing the Intersection of Race and Sex: A Black Feminist Critique of Antidiscrimination Doctrine, Feminist Theory, and Antiracist Politics’ (1989) Feminist Legal Theory 57.

32 Catharine MacKinnon, ‘Feminism, Marxism, Method, and the State: Toward Feminist Jurisprudence’ (1983) Feminist Legal Theory 181.

33 CAS 2019/A/6388, award of 14 July 2020

34 Ibid [207]

35 This term is no longer defined in the 2018 edition of the FCE. Therefore, The Panel in Karim looked to its common meaning in the English language.

36 CAS 2019/A/6388 Karim Keramuddin v. FIFA, award of 14 July 2020 [223]

37 Ibid [230]

38 Ibid [230]

39 Ibid

40 Ibid [231]

41 Ibid

42 Ibid [140]

43 Ibid [143]

44 Ibid [152]

45 Ibid [159]

46 Ibid [176]

47 Ibid [181]

48 Ibid [190]

49 Diane Gill, ‘A Feminist Perspective on Sport Psychology Practice’ (1994) 8(4) The Sport Psychologist 411

50 CAS 2011/A/2384 & CAS 2011/A/2386

51 CAS 2009/A/1920

52 Yvette Félix, Decision of the Adjudicatory Chamber of the Ethics Committee (16 March 2021)

53 Nella Joseph, Decision of the Adjudicatory Chamber of the Ethics Committee (23 April 2021)

